# Fluid balance and mortality in critically ill patients with acute kidney injury: a multicenter prospective epidemiological study

**DOI:** 10.1186/s13054-015-1085-4

**Published:** 2015-10-23

**Authors:** Na Wang, Li Jiang, Bo Zhu, Ying Wen, Xiu-Ming Xi, The Beijing Acute Kidney Injury Trial (BAKIT) Workgroup

**Affiliations:** Department of Critical Care Medicine, Fuxing Hospital, Capital Medical University, no.20 Fuxingmenwai Street, Xicheng District, Beijing, 100038 China; Emergency Department, China Rehabilitation Research Center, Capital Medical University, no.10 Jiaomen North Street, Fengtai District, Beijing, 100068 China

## Abstract

**Introduction:**

Early and aggressive volume resuscitation is fundamental in the treatment of hemodynamic instability in critically ill patients and improves patient survival. However, one important consequence of fluid administration is the risk of developing fluid overload (FO), which is associated with increased mortality in patients with acute kidney injury (AKI). We evaluated the impact of fluid balance on mortality in intensive care unit (ICU) patients with AKI.

**Methods:**

The data were extracted from the Beijing Acute Kidney Injury Trial. This trial was a prospective, observational, multicenter study conducted in 30 ICUs among 28 tertiary hospitals in Beijing, China, from 1 March to 31 August 2012. In total, 3107 patients were admitted consecutively, and 2526 patients were included in this study. The data from the first 3 sequential days were analyzed. The AKI severity was classified according to the Kidney Disease: Improving Global Outcomes guidelines. The daily fluid balance was recorded, and the cumulative fluid balance was registered at 24, 48, and 72 h. A multivariate analysis was performed with Cox regression to determine the impact of fluid balance on mortality in patients with AKI.

**Results:**

Among the 2526 patients included, 1172 developed AKI during the first 3 days. The mortality was 25.7 % in the AKI group and 10.1 % in the non-AKI group (*P* < 0.001). The daily fluid balance was higher, and the cumulative fluid balance was significantly greater, in the AKI group than in the non-AKI group. FO was an independent risk factor for the incidence of AKI (odds ratio 4.508, 95 % confidence interval 2.900 to 7.008, *P* < 0.001) and increased the severity of AKI. Non-surviving patients with AKI had higher cumulative fluid balance during the first 3 days (2.77 [0.86–5.01] L versus 0.93 [−0.80 to 2.93] L, *P* < 0.001) than survivors did. Multivariate analysis revealed that the cumulative fluid balance during the first 3 days was an independent risk factor for 28-day mortality.

**Conclusions:**

In this multicenter ICU study, the fluid balance was greater in patients with AKI than in patients without AKI. FO was an independent risk factor for the incidence of AKI and increased the severity of AKI. A higher cumulative fluid balance was an important factor associated with 28-day mortality following AKI.

**Electronic supplementary material:**

The online version of this article (doi:10.1186/s13054-015-1085-4) contains supplementary material, which is available to authorized users.

## Introduction

Early fluid resuscitation to expand intravascular volume and maintain organ perfusion is a core concept in the management of critical illness [1–4]. However, fluid administration increases the risk of fluid overload (FO). Clinically, FO manifests as an expansion of interstitial space and increased venous pressure, resulting in tissue edema, organ dysfunction [5–9], and adverse outcomes [10, 11].

Congestion and increased venous pressure lead to increased renal subcapsular pressure and lowered renal blood flow and glomerular filtration rate (GFR) [12]. The association between FO, the development of the abdominal compartment syndrome (ACS), and the occurrence of acute kidney injury (AKI) is well known [13]. Some investigators have observed that FO remained independently associated with adverse outcomes in patients with AKI after accounting for the confounding effects of illness severity and hemodynamic instability [14–16].

In a study done by the Program to Improve Care in Acute Renal Disease (PICARD) group, the adjusted odds ratio (OR) for mortality was 2.07 in patients with FO at initiation of renal replacement therapy (RRT). In this population, survivors who were taken off RRT showed significantly less FO than did patients who remained on RRT [17]. Recently, a reanalysis of the data from the Randomized Evaluation of Normal vs. Augmented Level of Replacement Therapy study demonstrated that, in patients with AKI requiring RRT in the intensive care unit (ICU), a negative mean daily fluid balance was independently associated with a decreased risk of death and with more ICU-free and hospital-free days [18]. FO is probably a severity marker or an independent factor for higher mortality in critically ill patients.

To date, there has been no multicenter study conducted on patients with AKI of Chinese descent. Thus, in our present study, we evaluated the impact of fluid balance on mortality in Chinese adult ICU patients with AKI.

## Material and methods

### Study design and data collection

**Fig. 1:**
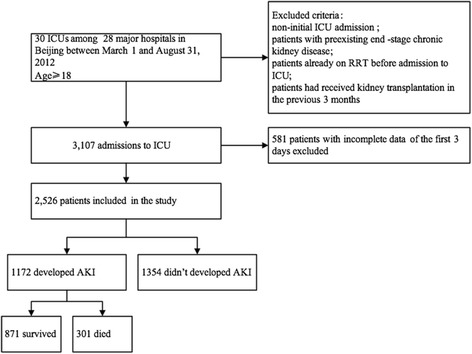
Study flowchart with 28-day mortality rate. *AKI* acute kidney injury, *ICU* intensive care unit, *RRT* renal replacement therapy

In this study, we used a database from a prospective, multicenter, observational study in which investigators examined the epidemiology of AKI in critically ill patients at 30 ICUs among 28 tertiary hospitals in Beijing, China, from 1 March to 31 August 2012 (the Beijing Acute Kidney Injury Trial [BAKIT]) [19]. For a complete list of these hospitals and the persons responsible for the data acquisition, see Additional file 1. Study subjects included all adult patients (age ≥18 years) admitted consecutively to ICUs. Only the initial ICU admission was considered in this study. The following patients were excluded: patients with preexisting end-stage chronic kidney disease, patients already undergoing RRT before admission to the ICU, and patients who had received kidney transplants in the previous 3 months. Preexisting comorbidities were diagnosed based on International Classification of Diseases, Tenth Revision, codes. Among the 3107 patients who were admitted consecutively, 2526 patients with their first 3 days of sequential data were included in our study (Fig. 1).

The collected data included demographics, anthropometrics, admission diagnosis, comorbidities, daily vital signs, and laboratory values, which were to calculate automatically the Acute Physiology and Chronic Health Evaluation II (APACHE II) [20], the Simplified Acute Physiology Score II (SAPS II) [21], and the Sequential Organ Failure Assessment (SOFA) score [22]. RRT and mortality data were also reported. Furthermore, hourly urine output, fluid balance, and use of diuretics were recorded daily. Fluid balance was calculated daily as the difference between fluid intake and fluid output. Fluid output included all body fluids, including urine, excrement, and, if applicable, dialysis ultrafiltrate. The patients were followed until death, hospital discharge, or for 28 days.

This study was approved by the institutional review board of the ethics committee of the lead study center (Fu Xing Hospital, Capital Medical University, Beijing China) and all other participating hospitals (see Additional file 2). The requirement for informed consent was waived for this observational survey. The patient records and information were anonymized and deidentified before analysis.

### Definitions

The AKI cohort consisted of patients who developed AKI within the first 72 h. Patients who developed AKI after the third day (e.g., on day 4) were classified as non-AKI. AKI severity was classified according to the Kidney Disease: Improving Global Outcomes (KDIGO) guidelines [23]. AKI was staged for severity (stages 1, 2, and 3) based on serum creatinine (SCr) or urine output or both. Baseline creatinine was defined as the lowest known SCr value during the previous 3 months [24]. For patients without these values or without renal failure, the baseline SCr was estimated by using the Modification of Diet in Renal Disease equation [25], assuming a GFR of 75 ml/min/1.73 m^2^ [26]. For patients with chronic renal failure but not receiving dialysis, the initial SCr value upon admission was used as the baseline value [26]. We regarded the worst stage during the first 3 days as the AKI stage.

Oliguria was defined as urine output less than 500 ml/day. The daily fluid balance was recorded, and the cumulative fluid balance was registered at 24, 48, and 72 h. We calculated the daily AKI numbers when we compared daily fluid balance between the AKI and non-AKI groups, but we included all patients with AKI in the first 3 days when we compared the cumulative fluid balance. To quantify the cumulative fluid balance over 3 days in relation to body weight, we used the following formula: sum of daily (fluid intake [liters] − total output [liters])/body weight (kilograms). We used the term *percentage of fluid accumulation* to define the percentage of cumulative fluid balance adjusted for body weight. Baseline body weight was recorded at initial hospital admission. We defined FO as fluid accumulation greater than 10 % of the baseline weight [27].

### Statistical analysis

Non-normally distributed continuous variables were expressed as median with interquartile range (IQR) and were compared using the Mann–Whitney *U* test or the Kruskal–Wallis analysis of variance test with the Bonferroni correction. Categorical variables were expressed as the number of cases and proportions and were compared using the Mantel–Haenszel χ^2^ test. We used a logistic regression model to evaluate the effect of FO on the incidence of AKI. We conducted exploratory univariate analysis of several variables to identify possible confounders associated with 28-day mortality and to assess the influence of cumulative fluid balance on the survival time of patients with AKI. A multivariate Cox regression analysis was then performed using a backward stepwise selection method, with a *P* value less than 0.05 as the entry criterion and a *P* value 0.10 or higher as the removal criterion. The assumption of proportional hazards was checked graphically using log (−log [survival probability) plots and was found to be appropriate. Variables considered for multivariable analysis included age, comorbid diseases, diagnosis, illness severity scores, cumulative fluid balance at 72 h, oliguria, and sepsis (similarly to a previous study) [28]. We tested for colinearity among all variables using a Cox regression analysis to generate hazard ratios and 95 % confidence intervals (CIs). The 28-day survival stratified by the presence or absence of FO was additionally evaluated graphically using the Kaplan–Meier product limit survival plot. All statistical analyses were performed using IBM SPSS 17.0 software package (IBM, Armonk, NY, USA), with a two-sided *P* value less than 0.05 considered statistically significant.

## Results

### Study population

Among the 3107 patients enrolled in the BAKIT study, 581 were excluded because of incomplete data covering the first 3 days following ICU admission, leaving 2526 patients for analysis. The characteristics of the entire cohort are shown in Table 1. The mean age was 64.0 (51.8–77.0) years, and 62.5 % were men. All-cause 28-day mortality was 17.3 %, and mean ICU length of stay was 6.0 (4.0–11.0) days. Of these patients, 1172 (46.4 %) developed AKI during the first 3 days following ICU admission. Patients with AKI were older (*P* < 0.001) and had higher illness severity scores than patients without AKI. Patients with AKI were more likely to present with sepsis upon ICU admission. The daily fluid balance and cumulative fluid balance at 24, 48, and 72 h were lower in the patients without AKI. Diuretics were used more commonly in patients with AKI (74.4 % versus 46.4 %; *P* < 0.001). The 28-day mortality was higher among patients with AKI (25.7 % versus 10.1 %; *P* < 0.001).

**Table 1:** Patient characteristics of all patients and compared between patients with and patients without acute kidney injury

Characteristics	All (n = 2526)	AKI (n = 1172)	Non-AKI (n = 1354)	*P* value
Age (yr)	64 (52–77)	67 (54–78)	63 (50–75)	<0.001
Male sex	1578 (62.5)	742 (63.3)	836 (61.7)	0.434
APACHE II score	14 (10–20)	17 (12–23)	13 (9–17)	<0.001
SAPS II score	34 (26–46)	40 (31–52)	31 (24–39)	<0.001
SOFA score	6 (4–9)	7 (5–10)	5 (3–7)	<0.001
Vasoactive therapy	1009 (40.0)	576 (49.1)	433 (32.0)	<0.001
Mechanical ventilation	1702 (67.4)	818 (69.8)	884 (65.3)	0.017
Baseline creatinine (μmol/L)	81.9 (69.0–96.0)	84.9 (73.0–97.0)	79.8 (66.8–95.0)	<0.001
Sepsis	808 (32.0)	512 (43.7)	296 (21.9)	<0.001
Fluid balance within 24 h (L)	0.57 (−0.19 to 1.43)	0.64 (−0.20 to 1.70)	0.53 (−0.18 to 1.30)	<0.001
Fluid balance within 48 h (L)	0.88 (−0.37 to 2.30)	1.06 (−0.38 to 2.82)	0.73 (−0.37 to 1.91)	<0.001
Fluid balance within 72 h (L)	1.12 (−0.48 to 2.86)	1.40 (−0.49 to 3.54)	0.91 (−0.48 to 2.44)	0.001
Use of diuretics (%)	1500 (59.4)	872 (74.4)	628 (46.4)	<0.001
28-day mortality	438 (17.3)	301 (25.7)	137 (10.1)	<0.001
Length of ICU stay (days)	6 (4–11)	7 (5–14)	5 (4–9)	<0.001

Data are expressed as median (interquartile range) or number (percent)

*AKI* acute kidney injury, *ICU* intensive care unit, *APACHE II* Acute Physiology and Chronic Health Evaluation II, *SAPS II* Simplified Acute Physiology Score II, *SOFA* Sequential Organ Failure Assessment

### Characteristics of patients with AKI

The characteristics of patients with AKI according to outcome are shown in Table 2. Non-surviving patients with AKI were older (*P* < 0.001), had higher illness severity scores, and were more likely to be diagnosed with sepsis than the other groups. The cumulative fluid balance during the first 3 days was higher among non-survivors (2.77 [0.86–5.01] L versus 0.93 [−0.80 to 2.93] L; *P* < 0.001). Oliguria was also more common in non-survivors (16.6 % versus 5.3 %; *P* < 0.001) than in patients with AKI who survived.

**Table 2:** Characteristics of patients with acute kidney injury, by outcome

Characteristics	AKI (n = 1172)	Survivors (n = 871)	Non-survivors (n = 301)	*P* value
Male sex	742 (63.3)	548 (62.9)	194 (64.5)	0.677
Age (yr)	67 (54–78)	64 (52–77)	74 (60–82)	<0.001
ICU admission				
APACHE II score	17 (12–23)	15 (11–21)	23 (17–29)	<0.001
SAPS II score	40 (31–52)	37 (28–47)	52 (41–64)	<0.001
SOFA score	7 (5–10)	7 (4–10)	9 (7–12)	<0.001
Vasoactive therapy	576 (49.1)	419 (48.1)	157 (52.2)	0.229
Mechanical ventilation	818 (69.8)	614 (70.5)	204 (67.8)	0.383
Baseline creatinine (μmol/L)	84.9 (73.0–97.0)	84.0 (72.1–97.0)	86.9 (75.0–97.8)	0.78
Sepsis	512 (43.7)	309 (35.5)	203 (67.4)	<0.001
Comorbid diseases				
Cancer	164 (14.0)	112 (12.9)	52 (17.2)	0.067
Hypertension	523 (44.6)	379 (43.5)	144 (47.8)	0.202
Cardiovascular	274 (23.4)	177 (20.3)	97 (32.2)	<0.001
Chronic kidney disease	111 (9.5)	79 (9.1)	32 (10.6)	0.493
Diabetes	240 (20.5)	173 (19.9)	67 (22.2)	0.407
Category of ICU admission diagnosis				
Respiratory	208 (17.7)	125 (14.4)	83 (27.6)	<0.001
Neurologic	178 (15.2)	134 (15.4)	44 (14.6)	0.781
Postsurgery	556 (47.4)	443 (50.8)	113 (37.5)	<0.001
Cardiovascular	233 (19.9)	170 (19.5)	63 (20.9)	0.615
ICU course				
Cumulative fluid balance in 3 days (L)	1.40 (−0.49 to 3.54)	0.93 (−0.80 to 2.93)	2.77 (0.86–5.01)	<0.001
Oliguria	96 (8.2)	46 (5.3)	50 (16.6)	<0.001
Use of diuretics (%)	872 (74.4)	635 (72.9)	237 (78.7)	0.047
Outcomes				
AKI stage				
1	496 (42.3)	424 (48.7)	72 (23.9)	
2	289 (24.7)	215 (24.7)	74 (24.6)	<0.001
3	387 (33.0)	232 (26.6)	155 (51.5)	
RRT	222 (18.9)	125 (14.4)	97 (32.2)	<0.001
Length of ICU stay (days)	7 (5–14)	7 (4–13)	8 (6–14)	0.001

Data are expressed as median (interquartile range) or number (percent)

*AKI* acute kidney injury, *SAPS II* Simplified Acute Physiology Score II, *SOFA* Sequential Organ Failure Assessment, *APACHE II* Acute Physiology and Chronic Health Evaluation II, *RRT* renal replacement therapy

### Fluid balance and the incidence of AKI

**Fig. 2:**
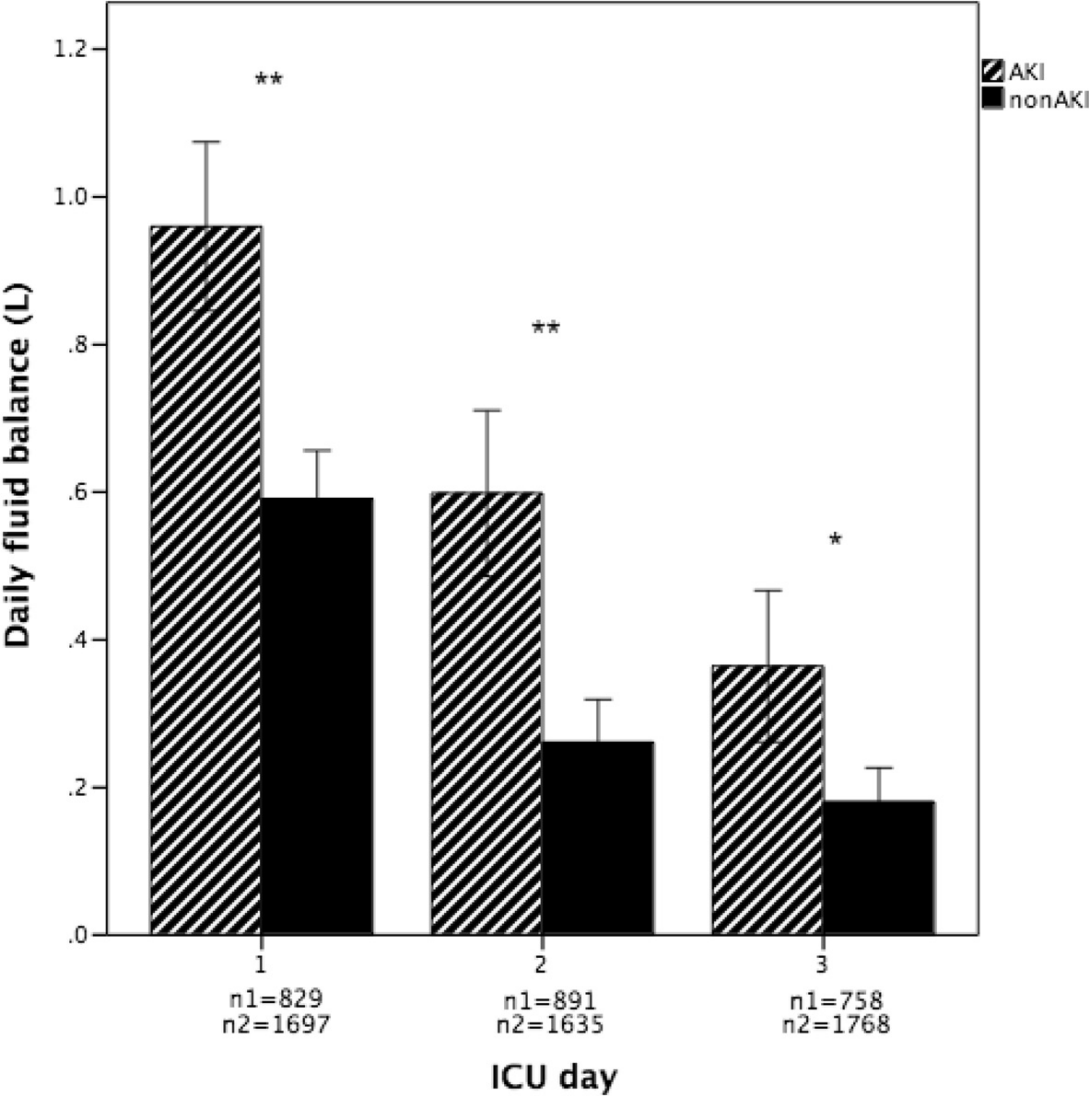
Daily fluid balance in acute kidney injury (AKI) and non-AKI in the first 3 days of intensive care unit (ICU) stay (mean ± standard error of the mean). *P = 0.007; **P < 0.001. n1 represents patients with AKI; n2 represents patients without AKI

**Fig. 3:**
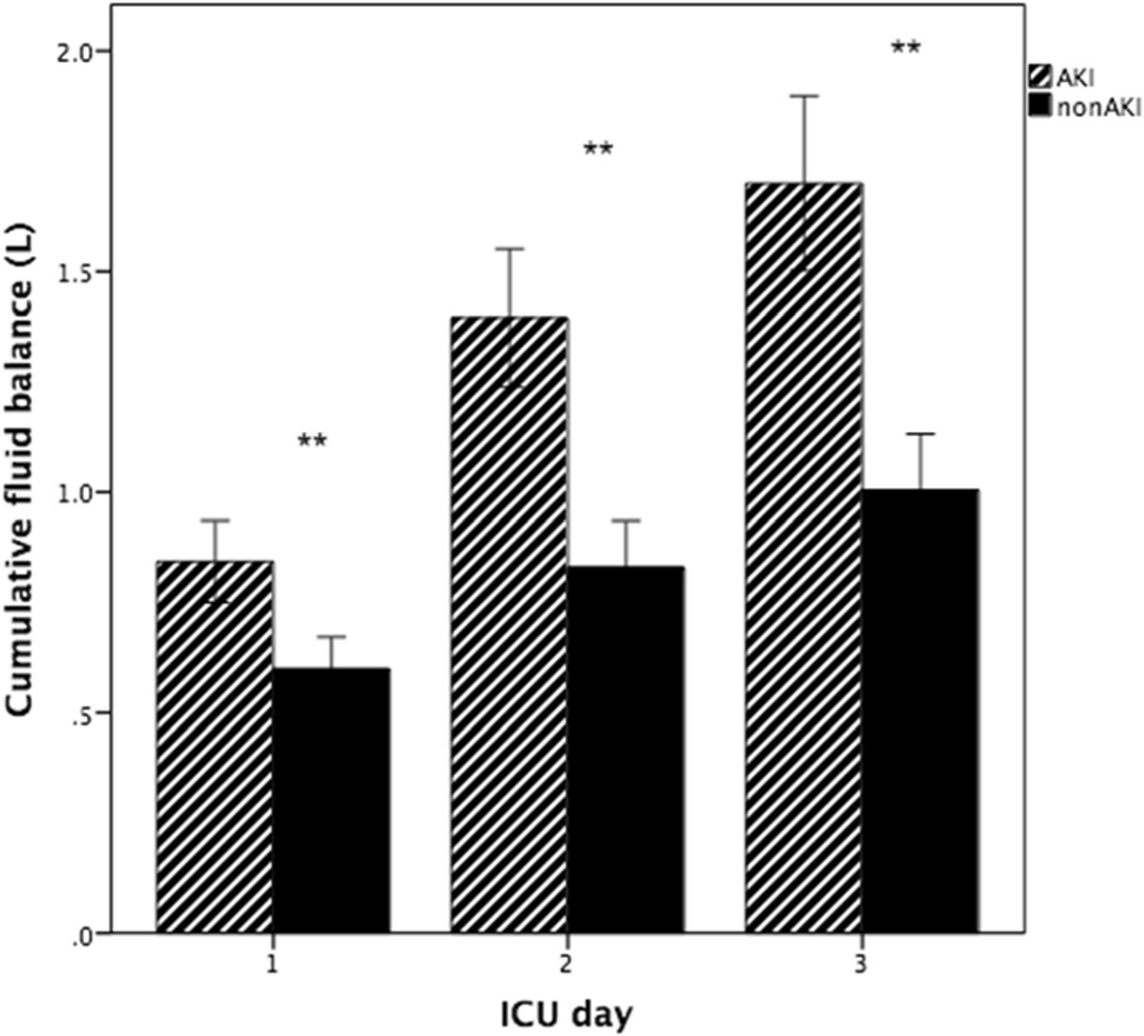
Cumulative fluid balance in acute kidney injury (AKI) and non-AKI at 24, 48, and 72 h of intensive care unit (ICU) stay (mean ± standard error of the). **P < 0.001

The patients with AKI manifested higher daily fluid balance than patients without AKI (*P* < 0.01) (Fig. 2). We included all patients with AKI during the first 3 days when comparing cumulative fluid balance, and patients with AKI consistently showed higher cumulative fluid balance at 24, 48, and 72 h following ICU admission than patients without AKI (Fig. 3).

We evaluated the effect of FO on the occurrence of AKI in the entire cohort. According to a multivariable model, FO increased the incidence of AKI distinctly (OR 4.508, 95 % CI 2.900–7.008, *P* < 0.001) (see Additional file 3: Table S1).

We classified the percentage of fluid accumulation into four levels: loss or negative, 0–5.0 % gain, 5.1–10 % gain, and greater than 10 % gain. We found that with the increase in fluid accumulation, the distribution of AKI severity was also changed. However, there was no significant difference between the first three groups. When the fluid volume was more than 10 %, the percentage of patients in AKI stage 1 decreased significantly and patients in AKI stage 3 increased significantly (*P* < 0.001) (see Additional file 3: Figure S1).

### Fluid balance and the mortality of patients with AKI

**Fig. 4:**
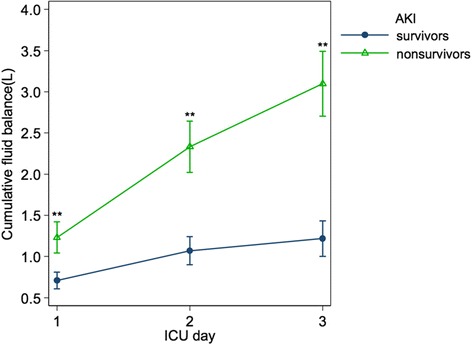
Cumulative fluid balance in acute kidney injury (AKI) survivors and non-survivors in the first 3 days of their intensive care unit (ICU) stay (mean ± standard error of the mean). **P < 0.001

Our study demonstrated the pattern of cumulative fluid balance in survivors and non-survivors among patients with AKI during the first 3 days in the ICU (Fig. 4). The cumulative fluid balance over 3 days among the AKI survivors was 0.93 (−0.80 to 2.93) L and 2.77 (0.86–5.01) L (*P* < 0.001) in the AKI non-survivors. Non-survivors also showed higher cumulative fluid balance at 24, 48, and 72 h with significant differences.

**Fig. 5:**
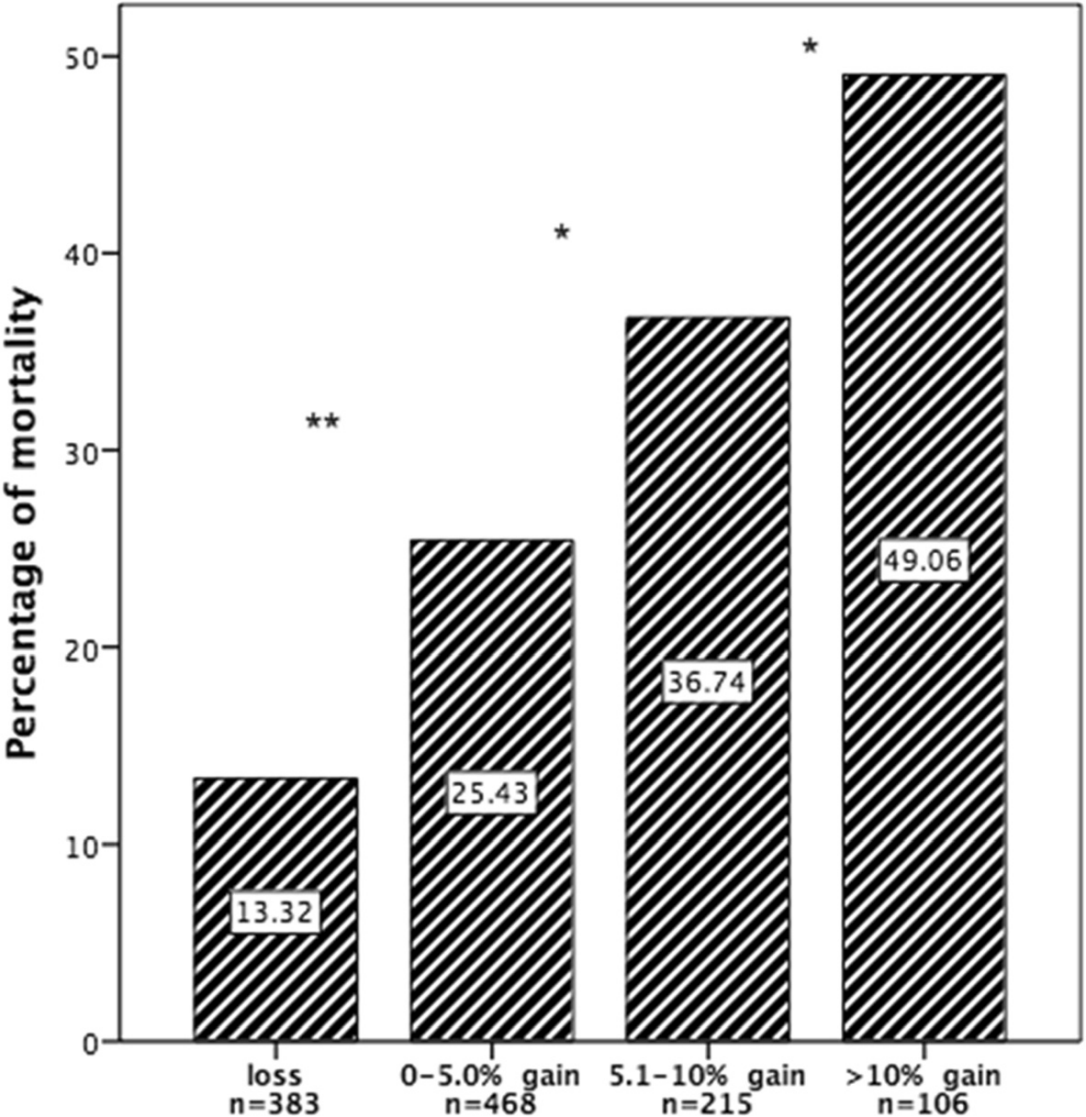
Mortality rate by fluid accumulation in 3 days relative to baseline weight in patients with acute kidney injury. *P* value is the result of comparing the neighboring groups. *P < 0.05; **P < 0.001

When we stratified the patients with AKI by fluid accumulation in 3 days relative to baseline weight (Fig. 5), the mortality increased among patients with more accumulated fluid. The mortality of patients with negative fluid balance was the lowest.

**Fig. 6:**
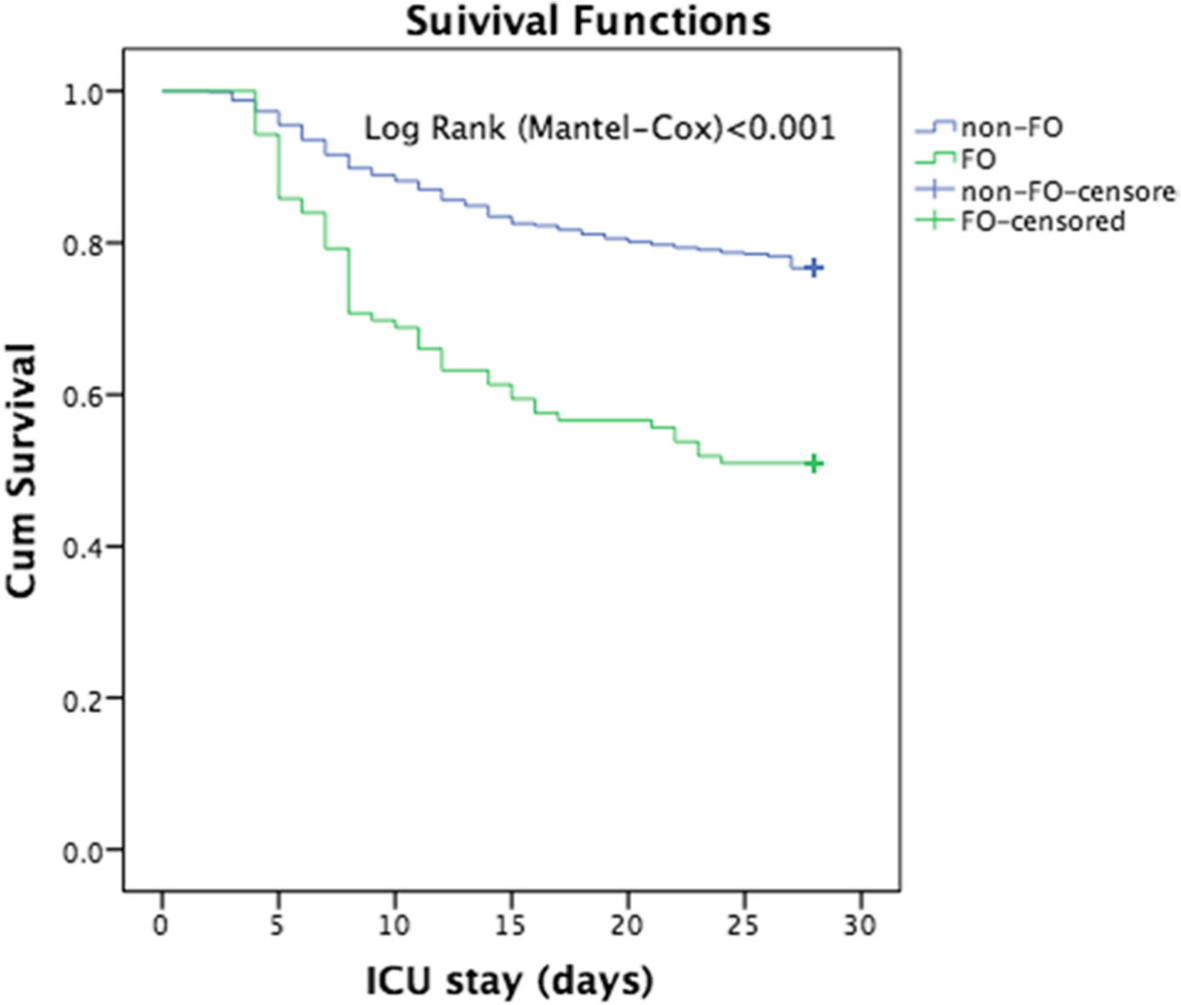
Survival curve of 28-day mortality by the presence or absence of fluid overload (FO) in the patients with acute kidney injury in the intensive care unit (ICU)

In the multivariate Cox regression analysis (Table 3), age, SAPS II score, cumulative fluid balance over 3 days, RRT, and sepsis were independent predictors of 28-day mortality. The presence of FO was associated with a higher risk of death (Fig. 6). When FO occurred, the mortality of patients without AKI and those with AKI increased from 9.6 % to 33.3 % (*P* = 0.001) and from 23.4 % to 49.1 %, respectively (*P* < 0.001) (see Additional file 3: Figure S2).

**Table 3:** Multivariate Cox regression analysis of 28-day mortality in critically ill patients with acute kidney injury

Characteristic	Hazard ratio	95 % CI	*P* value
Age	1.013	1.005–1.020	0.002
SAPS II	1.021	1.014–1.029	<0.001
Cumulative fluid balance in 3 days	1.041	1.012–1.072	0.006
Sepsis	1.278	0.961–1.701	0.092
Postsurgery	0.760	0.595–0.971	0.028
CRRT	3.166	2.463–4.069	<0.001

*CI* confidence interval, *CRRT* continuous renal replacement therapy, *SAPS II* Simplified Acute Physiology Score II

## Discussion

We investigated the influence of fluid balance on outcomes among critically ill patients with AKI in a large, multicenter, observational study involving 30 ICUs. Patients who developed AKI were older, more severely ill, and more frequently presented with sepsis upon ICU admission than patients without AKI. The mortality rates were higher among patients with AKI than in those without AKI, particularly in patients with oliguria. The daily fluid balance was higher in the AKI group than in the non-AKI group. Patients with AKI consistently had higher cumulative fluid balance at 24, 48, and 72 h following ICU admission than patients without AKI. The cumulative fluid balance over 3 days was significantly higher among non-surviving patients with AKI and was significantly associated with increased 28-day mortality in patients with AKI.

The fluid balance was higher in patients with AKI. According to the multivariable model, the presence of FO increased the incidence of AKI distinctly. However, we could not determine the relationship between a positive fluid balance and the incidence of AKI, because we failed to determine the fluid balance before ICU admission, which might have influenced the incidence of AKI and outcomes. The findings of the present study should stimulate further research to investigate the role of fluid balance on the incidence of AKI.

In fact, several lines of evidence suggest that fluid therapy, rather than preserving renal function, actually precipitates or worsens AKI by causing FO. Positive fluid balance triggers AKI after cardiac surgery [29, 30]. On the contrary, the use of goal-directed therapy (GDT) strategies for perioperative hemodynamic optimization have been associated with decreased surgical complications [31–33] and reduced risk of postoperative AKI [34]. Prowle et al. reviewed 24 studies and found that GDT in surgery was associated with a significantly lower incidence of AKI (OR 0.59, 95 % CI 0.39–0.89, *P* = 0.013; n = 24 studies, n = 2763 patients) [35].

In our study, FO affected the incidence of AKI and the severity of AKI. AKI severity increased significantly when the fluid volume was more than 10 %. The degree of FO has also been suggested as an index of AKI severity in pediatric patients [36]. However, the cutoff value is not clear, because an elaborate ROC curve analysis would be required.

In our study, fluid accumulation was associated with adverse outcomes in patients with AKI, and this finding is consistent with those of prior studies. Teixeira et al. performed a secondary analysis of data from a multicenter, prospective cohort study in 10 Italian ICUs [28] including 601 patients. They found that mean fluid balance (MFB) was higher (*P* = 0.008) in patients with AKI. When analyzing the subgroups separately by 28-day mortality, non-survivor patients with AKI had significantly higher MFB than survivors. In a multicenter, prospective, observational study with 296 patients from 17 ICUs, Vaara et al. also reported that FO in renal support therapy (RST) was associated with a higher risk of death at 90 days (OR 2.6) after adjustment for severity of illness, RST onset time, RST modality, and sepsis [37]. The Sepsis Occurrence in Acutely Ill Patients study investigators found that FO in septic patients with AKI was associated with higher mortality at 60 days [15]. Several other studies also showed positive fluid balance increased mortality among ICU patients [38–41].

The relationship of fluid accumulation and mortality associated with AKI is complex. It is not possible to determine whether the positive fluid balance found in patients with AKI was the cause or the result of a greater severity of illness. Perhaps there was a higher severity of illness and hypotension among those who received more fluids, which are well-known clinical risk factors for AKI and mortality. There are many factors that affect the prognosis of patients with AKI, and we needed to rule out confounding factors and perform a propensity analysis to further explore this issue.

When we stratified the patients with AKI by fluid accumulated during the first 3 days, we found that greater fluid accumulation increased mortality among patients with AKI. In contrast, the mortality of patients with a negative fluid balance was the lowest. Our findings are in agreement with reports of a number of previous studies. Bouchard et al. [17] evaluated the adult population with AKI in the PICARD study and found that, at the time of AKI diagnosis, the percentage of fluid accumulation in relation to the patient’s weight upon ICU admission was lower among survivors than non-survivors (*P* = 0.01). When the rate of fluid accumulation of all patients was greater than 10 %, the mortality at 30 and 60 days climbed from 25 % to 37 % (*P* = 0.02) and from 35 % to 48 % (*P* = 0.01), respectively. Patients who maintained fluid accumulation during their hospitalization showed higher mortality proportional to fluid buildup (*P* < 0.001). Vaara et al. [37] demonstrated a direct association between cumulative FO at RRT initiation and an increased risk of 90-day mortality. Researchers in a pediatric study also found a 3 % increase in mortality for every 1 % increase in FO. Children with more than 20 % FO had an OR for mortality of 8.5 compared with children with less than 20 % FO [42].

RRT might be effective in reducing FO and increasing survival. However, in our study, RRT was an independent risk factor for 28-day mortality (Table 3). The mortality of patients treated with RRT was higher (see Additional file 3: Table S2). This finding may be explained partly by increased illness severity upon ICU admission. Our findings in this regard are consistent with those of other studies [15, 43].

This study is unique in providing detailed insights into fluid balance and mortality in critically ill patients with AKI in Beijing, China. Compared with previous studies, we defined and classified AKI severity according to the KDIGO criteria, reducing underestimation or late recognition of AKI. In addition, the study subjects with 3-day sequential data included in our study may decrease selection bias, and the results are thus more credible than in other studies. Our study provides insight into the significance of fluid accumulation in terms of degree and duration.

However, some limitations must be considered. First, fluid balance before ICU admission was not measured, which might influence the incidence of AKI and the outcomes. Second, fluid gain could be the result of either overzealous fluid therapy or poor urine output; we could not differentiate between the two components. Third, there are many factors that affect the prognosis of patients with AKI. Fluid balance is one of these factors, and we need to perform a propensity analysis to further explore this issue. In addition, we failed to determine the type of fluid given (i.e., colloid versus crystalloid, parenteral versus enteral), aside from the volume, which may have influenced outcomes. We also excluded patients who had been in the ICU for fewer than 3 days but had more severe illness, and our results could have been a little more meaningful if we had included such patients.

## Conclusions

This large, multicenter, observational study confirms that higher fluid balance was an important factor associated with 28-day mortality among patients with AKI. Fluid balance was higher in the AKI group than in the non-AKI group. FO was an independent risk factor for the incidence of AKI and increased the AKI severity. Further studies are needed to investigate the mechanisms underlying the role of fluid balance in patients with AKI.

## Key messages

- In this multicenter ICU study, fluid balance was higher in the AKI group than in the non-AKI group.
- FO was an independent risk factor for the incidence of AKI and increased AKI severity.
- A higher cumulative fluid balance was an important factor associated with 28-day mortality following AKI.

## Additional files

Additional file 1:
**Members of the Beijing Acute Kidney Injury Trial (BAKIT) workgroup.** (DOC 30 kb) Additional file 2:
**All other ethical bodies that approved our study in the various centers involved.** (DOC 25 kb) Additional file 3: Table S1. Logistic regression analysis of AKI incidence in critically ill patients. OR, odds ratio; CI, confidence interval; APACHE II, Acute Physiology and Chronic Health Evaluation II; SAPS II, Simplified Acute Physiology Score II; SOFA, Sequential Organ Failure Assessment; FO, fluid overload. **Table S2.** Characteristics of patients with AKI, stratified by treatment with or without RRT. Data are expressed as median (interquartile range) or number (percentage). AKI, acute kidney injury; RRT, renal replacement therapy; APACHE II, Acute Physiology and Chronic Health Evaluation II; SAPS II, Simplified Acute Physiology Score II; SOFA, Sequential Organ Failure Assessment. **Figure S1.** Percentage of AKI stages by fluid accumulation in 3 days relative to baseline weight in patients with AKI. *P* value represents comparison of the neighboring groups. **P* >0.05; ***P* <0.001. **Figure S2.** Mortality of FO/Non-FO by the presence or absence of AKI in the overall patients in the ICU. FO, fluid overload. **P* =0.001; ***P* <0.001. (DOC 142 kb)

## Abbreviations

AKI: acute kidney injury
APACHE II: Acute Physiology and Chronic Health Evaluation II
BAKIT: Beijing Acute Kidney Injury Trial
CI: confidence interval
CRRT: continuous renal replacement therapy
FO: fluid overload
GDT: goal-directed therapy
GFR: glomerular filtration rate
ICU: intensive care unit
IQR: interquartile range
KDIGO: Kidney Disease: Improving Global Outcomes
MFB: mean fluid balance
PICARD: Program to Improve Care in Acute Renal Disease
RRT: renal replacement therapy
RST: renal support therapy
SAPS II: Simplified Acute Physiology Score II
SCr: serum creatinine
SOFA: Sequential Organ Failure Assessment
