# Supplementary material for: Fluid balance and mortality in critically ill patients with acute kidney injury: a multicenter prospective epidemiological study
Source: Crit Care. 2015 Oct 23;19:371. doi: 10.1186/s13054-015-1085-4 (PMC4619072; doi:10.1186/s13054-015-1085-4)
Supplement: Additional file 2: — All other ethical bodies that approved our study in the various centers involved. (DOC 25 kb) [file 13054_2015_1085_MOESM2_ESM.doc]

### *Additional file 2:*

**All other ethical bodies that approved our study in the various centers involved:**

The institutional review board of Peking Union Medical College Hospital

The institutional review board of Beijing Tongren Hospital, Capital Medical University

The institutional review board of Beijing Tiantan Hospital affiliated to Capital Medical University

The institutional review board of Beijing Friendship Hospital, Capital Medical University

The institutional review board of Beijing Ditan Hospital , Capital Medical University

The institutional review board of Beijing Chaoyang Hospital, Capital Medical University

The institutional review board of Xuanwu Hospital, Capital Medical University

The institutional review board of Peking University Third Hospital

The institutional review board of Peking University First Hospital

The institutional review board of Peking University People’s Hospital

The institutional review board of China-Japan Friendship Hospital

The institutional review board of The 309th Hospital of Chinese People’s Liberation Army

The institutional review board of Beijing Shijitan Hospital, Capital Medical University

The institutional review board of Fuwai Hospital, China Academy of Medical Science and Peking Union Medical College

The institutional review board of Air Force General Hospital of Chinese People’s Liberation Army

The institutional review board of The First Affiliated Hospital of General Hospital of People’s Liberation Army

The institutional review board of Navy General Hospital

The institutional review board of The Luhe Teaching Hospital of the Capital Medical University

The institutional review board of Beijing Anzhen Hospital, Capital Medical University

The institutional review board of Beijing Hospital

The institutional review board of General Hospital of Armed Police Forces

The institutional review board of The General Hospital of People’s Liberation Army

The institutional review board of Beijing YouAn Hospital, Capital Medical University

The institutional review board of HuaXin Hospital, First Hospital of Tsinghua University

The institutional review board of Beijing Shunyi Hospital of China Medical University

The institutional review board of Beijing Geriatric Hospital

The institutional review board of Beijing No.6 Hospital
