# Supplementary material for: Fluid balance and mortality in critically ill patients with acute kidney injury: a multicenter prospective epidemiological study
Source: Crit Care. 2015 Oct 23;19:371. doi: 10.1186/s13054-015-1085-4 (PMC4619072; doi:10.1186/s13054-015-1085-4)
Supplement: Additional file 3: Table S1. — Logistic regression analysis of AKI incidence in critically ill patients. OR, odds ratio; CI, confidence interval; APACHE II, Acute Physiology and Chronic Health Evaluation II; SAPS II, Simplified Acute Physiology Score II; SOFA, Sequential Organ Failure Assessment; FO, fluid overload. Table S2. Characteristics of patients with AKI, stratified by treatment with or without RRT. Data are expressed as median (interquartile range) or number (percentage). AKI, acute kidney injury; RRT, renal replacement therapy; APACHE II, Acute Physiology and Chronic Health Evaluation II; SAPS II, Simplified Acute Physiology Score II; SOFA, Sequential Organ Failure Assessment. Figure S1. Percentage of AKI stages by fluid accumulation in 3 days relative to baseline weight in patients with AKI. P value represents comparison of the neighboring groups. *P >0.05; **P <0.001. Figure S2. Mortality of FO/Non-FO by the presence or absence of AKI in the overall patients in the ICU. FO, fluid overload. *P =0.001; **P <0.001. (DOC 142 kb) [file 13054_2015_1085_MOESM3_ESM.doc]

### *Additional file 3:*

Additional Tables and Figures:

| TableS1 Logistic regression analysis of AKI incidence in critically ill patients | | | |  |
| --- | --- | --- | --- | --- |
| **Characteristic** | **OR** | **95%CI** | **P** |  |
| **APACHEII** | 1.019 | 1.001--1.039 | 0.043 |  |
| **SAPS II** | 1.010 | 1.001--1.019 | 0.035 |  |
| **SOFA** | 1.168 | 1.133--1.204 | 0.000 |  |
| **Sepsis** | 1.324 | 1.082-1.620 | 0.006 |  |
| **FO** | 4.508 | 2.900-7.008 | 0.000 |  |

OR,Odds Ratio;CI, confidence interval; APACHE II, Acute Physiology and Chronic Health Evaluation II; SAPS II, Simplified Acute Physiology Score II.;SOFA, sequential organ failure assessment; FO, fluid overload.

| Table S2 Characteristics of patients with AKI, stratified by treatment with or without RRT | | | |
| --- | --- | --- | --- |
|  | **RRT**  **(n=222)**  **Median(IQR)**  **Number (%)** | **Non－RRT**  **(n=950)**  **Median(IQR)**  **Number (%)** | **P**  **value** |
| Age(years) | 66（52-79） | 67（54-78） | 0.64 |
| Male gender | 148(66.7) | 594(62.5) | 0.28 |
| APACHEII | 23(17 to 29) | 16(11to 22) | <0.001 |
| SAPSII | 48(38 to 62) | 38(29 to 50) | <0.001 |
| SOFA | 10(7-13) | 7(5-9) | <0.001 |
| Vasoactive therapy | 121(54.5) | 455(47.9) | 0.086 |
| Sepsis | 512（43.7） | 296（21.9） | <0.001 |
| 28-day mortality | 97（43.7） | 204（21.5） | <0.001 |
| Length of ICU stay (days) | 11(7-19) | 7(4-12) | <0.001 |

Data are expressed as median (interquartile range),number (percentage). AKI, acute kidney injury; RRT, renal replacement therapy; APACHE II, Acute Physiology and Chronic Health Evaluation II; SAPS II, Simplified Acute Physiology Score II; SOFA, sequential organ failure assessment.


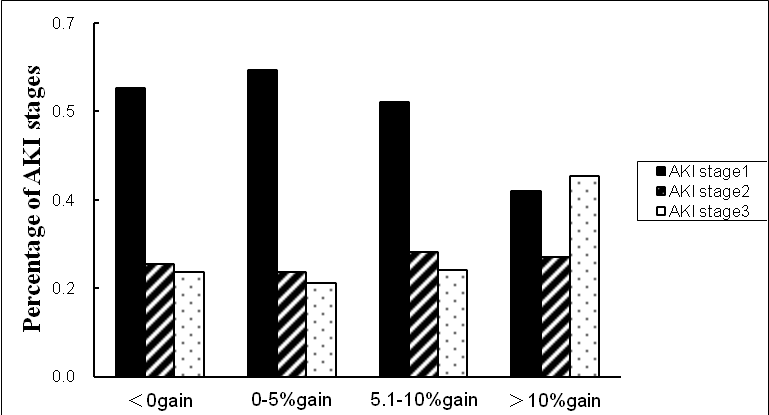


Figure S1 Percentage of AKI stagesby fluid accumulation in 3 days relative to baseline weight in AKI patients. P value is the comparing result of the neighboring groups.*P >0.05; **P <0.001.


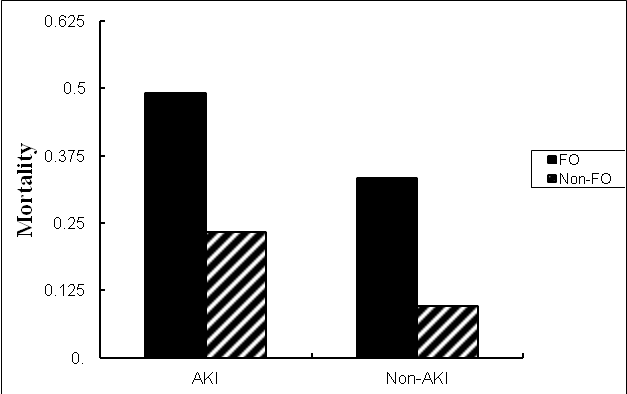


Figure S2 Mortality of FO/Non-FO by the presence or absence of AKI in the overall patients in the ICU. FO, fluid overload.*P =0.001; **P <0.001.
